# Effect of Perioperative *β*-Blockers on Pulmonary Complications among Patients with Chronic Obstructive Pulmonary Disease Undergoing Lung Resection Surgery

**DOI:** 10.1155/2015/204826

**Published:** 2015-09-01

**Authors:** A. Kamath, D. E. Stover, A. Hemdan, I. Belinskaya, R. M. Steingart, Y. Taur, M. B. Feinstein

**Affiliations:** ^1^Long Island Pulmonary and Sleep Associates, 200 North Village Avenues, Suite 300, Rockville Centre, NY 11750-5294, USA; ^2^Primary Research Institution, Memorial Sloan-Kettering Cancer Center, 1275 York Avenue, New York, NY 10021-9809, USA

## Abstract

The aim of this study is to determine if COPD patients undergoing lung resection with perioperative *β*-blocker use are more likely to suffer postoperative COPD exacerbations than those that did not receive perioperative *β*-blockers. *Methods.* A historical cohort study of COPD patients, undergoing lung resection surgery at Memorial Sloan-Kettering Cancer Center between 2002 and 2006. Primary outcomes were the rate of postoperative COPD exacerbations, defined as any initiation or increase of glucocorticoids for documented bronchospasm. *Results.* 520 patients with COPD were identified who underwent lung resection. Of these, 205 (39%) received perioperative *β*-blockers and 315 (61%) did not. COPD was mild among 361 patients (69% of all patients), moderate in 117 patients (23%), and severe in 42 patients (8%). COPD exacerbations occurred among 11 (5.4%) patients who received perioperative *β*-blockers and among 20 (6.3%) patients who did not. Secondary outcomes, which included respiratory failure, 30-day mortality, and the presence or absence of any cardiovascular complication, ICU transfer, cardiovascular complication, or readmission within 30 days, did not differ in prevalence between the two groups. *Conclusions.* This study implies that perioperative *β*-blockers use among COPD patients undergoing lung resection surgery does not impact the rate of exacerbations.

## 1. Introduction

An estimated 71% of patients with non-small-cell lung cancer undergo potentially curative lung cancer resection in the United States every year [1]. Other than the lung, the heart is the organ most likely to suffer complications from lung resection [2]. Cardiac complications, which can include acute coronary syndrome, atrial fibrillation, and/or tachyarrhythmias, may prolong hospitalization and increase the overall financial cost of surgery [2–7]. The risk of cardiac complications can be decreased among some patients at high risk for acute coronary syndrome by the use of perioperative *β*-blockers. Mangano and colleagues, for instance, found that the use of atenolol, a *β*_1_ selective agent, was associated with improved mortality among 200 V.A. patients with greater than two risk factors for coronary artery disease [8].

Unfortunately, *β*-blockers, especially those that bind nonselectively, have the potential to block the *β*_2_ receptors of airway epithelium. Concern of precipitating bronchospasm in this fashion has led many investigators to exclude patients with chronic obstructive pulmonary disease (COPD) from clinical trials involving *β*-blockers. Such concerns may not be entirely justified [9]. Salpeter et al. demonstrated in a Cochrane review that 29 of 30 patients with asthma or COPD sufficiently severe to warrant chronic bronchodilators safely tolerated therapeutic levels of *β*-blockade with metoprolol or atenolol [10]. Au et al. demonstrated that *β*-blocker use among 1,966 patients with COPD and hypertension was associated with a lower all-cause mortality than use of other antihypertensives [11]. Finally, Çamsari et al. found that metoprolol given for coronary artery disease did not decrease the forced expiratory volume in one second (FEV1) among 50 patients with stable moderate to severe COPD [12].

Due to a common risk factor, smoking, many patients undergoing thoracic resection may suffer from heart disease and COPD concurrently [13–17]. As such, they represent a unique population who may benefit from the cardioprotective properties of *β*-blockers while concurrently being at increased risk for bronchospasm from the same medications. To our knowledge, all previously published studies of *β*-blocker use among COPD patients undergoing lung resection surgery have been case reports or lacked adequate controls. This study endeavored to include control patients to quantitate the potential risks and benefits of *β*-blocker use; its aim is to determine whether patients receiving perioperative *β*-blockers were more likely to suffer postoperative COPD exacerbations than those who did not.

## 2. Methods

We conducted a historical cohort study of COPD patients, undergoing lung resection surgery at Memorial Sloan-Kettering Cancer Center between 2002 and 2006 (IRB approval number WA0264-04). Billing codes were used to identify patients who had undergone both pulmonary function tests and thoracic surgery at our institution. The PFTs for each individual patient were extractable electronically. All patients met the ATS criteria for COPD. COPD was defined as the ratio for forced expiratory volume in one second to forced vital capacity (FEV1/FVC) being less than or equal to 70% of predicted. Mild COPD was defined as having a percent predicted forced expiratory volume in one second (FEV1%) greater than or equal to 80%. Moderate COPD was defined as having a predicted FEV1% between 50% and less than 80%. Severe COPD was defined as having a predicted FEV1% as less than 50% [18].

While the type and dose of *β*-blocker that each patient received was initially derived electronically from the medical record, a chart review was performed for each patient to confirm the dose and to delineate the patients who received *β*-blockers chronically from those who received only the perioperative dose. No dedicated pharmacy database was needed at our institution, as drug information is also available in detail using the general electronic medical record.

Patients who received *β*-blockers in response to postoperative cardiac events including tachyarrhythmia or acute coronary syndrome were excluded. Data derived from the medical record included patient age and sex; type of surgery (described as wedge resection, segmentectomy, lobectomy, or pneumonectomy); history of heart disease; spirometry, including measurement of the forced expiratory volume in one second, expressed as percent predicted (FEV1%); diffusing capacity for carbon monoxide, expressed as percent predicted (DLCO%); length of hospital stay in days; mortality at 30 days; the presence or absence of a postoperative ICU stay; and the presence or absence of any cardiovascular complications as defined in the following. Among patients who received *β*-blockers perioperatively, the specific drug, the dose, and the manner of delivery were also recorded.

The study was completed prior to the revised ACA/AHA 2014 guidelines; however, it would have been unusual to first start a *β*-blocker on the day of surgery. Perioperative *β*-blockade may have been started at the earliest three or so days before surgery based on individual prescribing practices. Intravenous dosing of metoprolol (5 mg) was noted to be given on an as needed basis in the pre- and intraoperative period, to keep the heart rate and blood pressure within parameters set by the ordering physician. Postoperatively, oral *β*-blockers are resumed with a divided dose equivalent of metoprolol 50–100 mg.

Included in primary outcomes is the presence or absence of a postoperative COPD exacerbation. An exacerbation was considered to have occurred if systemic glucocorticoids were prescribed for documented bronchospasm or if a preexisting systemic steroid dose was increased by the equivalent of 20 mg of prednisone or more before hospital discharge. Patients receiving glucocorticoids for other causes were not considered to have suffered a COPD exacerbation. Secondary outcomes included the following: respiratory failure, 30-day mortality, and the presence or absence of any cardiovascular complication, ICU transfer, or readmission within 30 days. Respiratory failure was considered to have occurred when mechanical ventilation was necessary for more than 48 hours after thoracic surgery. A cardiovascular complication was defined as the development of acute coronary syndrome (ACS), congestive heart failure (CHF), and/or atrial fibrillation.

## 3. Statistics

Patients who had received perioperative *β*-blockers were compared to those who had not. Patients receiving *β*-blockers in response to an unexpected postoperative cardiac event, such as tachyarrhythmia or acute coronary syndrome, were not included in the *β*-blocker group. These predictors were first analyzed separately in a univariate model. To assess for confounding effects, variables with a univariate *p* value of 0.25 or lower were included in a simultaneous multivariate model. Administration of perioperative antibiotics was included in the multivariate model regardless of univariate significance. A similar strategy was employed for secondary outcomes.

## 4. Results

Between 2002 and 2006, 520 patients who met the ATS criteria for COPD and underwent lung resection surgery at our institution were identified. Of these, 205 (69.8%) patients received perioperative *β*-blockers and 315 (35.9%) did not. Demographic information is summarized on Table 1.

Among all patients, COPD was mild among 361 (69%), moderate in 117 (23%), and severe among 42 (8%). Patients prescribed *β*-blockers were slightly older (70 years old versus 67 years old (38% versus 9%; *p* < 0.0001)). The extent of surgical resection, as well as the median FEV1% and DLCO%, was not different between the two groups. Among those prescribed *β*-blockers, 111 (54%) patients had been receiving *β*-blocker medications chronically and 94 (46%) received them only for the perioperative period. The incidence of cardiac complications which included acute coronary syndrome, atrial fibrillation, CHF, and others not specified was not statistically significant between the two groups. The composite cardiac endpoints were 34 (16.6%) in the those patients receiving perioperative *β*-blockers versus 52 (16.5%) in those patient that did not, as shown in Table 2. *β*_1_ selective agents, such as metoprolol and atenolol, were prescribed much more commonly than nonselective agents, such as propranolol, labetalol, and carvedilol, as shown in Table 3. The use of noncardioselective *β*-blockers is suboptimal. However, this occurred at the discretion of the prescribing practice of individual physicians and not as a result of hospital protocol.

Overall, 5 patients (1%) died within 30 days of surgery. Although more of these patients were in the group receiving *β*-blockers, the small number of events precluded any meaningful statistical analysis.

The primary outcome, COPD exacerbation, occurred following less than 10% of surgical procedures. The rate of COPD exacerbations did not differ between patients receiving and not receiving perioperative *β*-blockers (*p* = 0.60). The secondary outcomes of respiratory failure, 30-day mortality, and the presence or absence of any cardiovascular complication, ICU transfer, or readmission within 30 days each occurred among less than 10% of surgical procedures. No significant differences in the rates of these complications were noted between patients who received *β*-blockers and those who did not (data not shown). Clinical predictors of COPD exacerbation were assessed using univariate and multivariate logistic regression, as shown in Table 4.

Variables were identified prospectively and included age >60 years, male sex, whether or not perioperative *β*-blocker were given, FEV1% <50, DLCO% <50, asthma history, cardiac disease history, chronic renal insufficiency (CRI), diabetes mellitus, smoking within 6 months, and the extent of lung tissue resected (sublobectomy, lobectomy, or bilobectomy and pneumonectomy). On univariate analysis, clinical predictors associated with COPD exacerbation following lung resection as noted on Table 4 included the following: FEV1 <50%, DLCO <50%, and smoking within six months. In multivariate analysis, only DLCO% remained as an independent predictor. Use of perioperative *β*-blockers was not associated with COPD exacerbation in either univariate or multivariate analysis.

## 5. Discussion

Results of this study imply that perioperative *β*-blockade is generally safe at lung resection surgery. The Dutch Echocardiographic Cardiac Risk Evaluation Applying Stress EKG (DECREASE) trial, reporting a 90% relative risk reduction in the combined endpoint of postoperative cardiac death and nonfatal myocardial infarction, included fabricated data and should, therefore, be discredited [19–22]. This was further challenged by the Perioperative Ischemic Evaluation (POISE) trial, a far superior randomized control trial, which showed an increased mortality and stroke with high dose perioperative *β*-blockade [23]. A more recent scientific review of the data by the American College of Cardiology and the American Heart Association in 2014 revised the clinical practice perioperative guidelines and should be taken into consideration when contemplating the use of perioperative beta-blockers. A class I B recommendation was given for the continuation of chronic *β*-blocker use perioperatively, a class II B recommendation was given for consideration of use in intermediate to high risk cardiac patients, and a class III B recommendation states that beta-blockers should not be started on the day of surgery [24]. This study found that acute coronary syndrome occurred in 1 (0.5%) of patients who received *β*-blockers in comparison to 0 (0%) in patients who did not, the results of which are not statistically significant. Predictors of COPD exacerbation among patients receiving *β*-blockers included age greater than 60 years, FEV1 < 50%, DLCO < 50%, and any history of heart disease.

This study found that COPD exacerbations developed in about 6% of the patients, which is lower than that found by some other investigators. While Leo et al. noted a 12% rate [25], any perceived difference may result from differences in how COPD exacerbation was defined. Leo defined it by the absence of other pulmonary complications and the concomitant presence of three of the following five signs: worsening dyspnea, purulent sputum, bronchial secretion volume >10 mL/24 h, fever without apparent cause, and wheezing. The definition of COPD exacerbation used in this study mandated the use of glucocorticoids for documented bronchospasm, which, while being more intuitive, may have missed milder exacerbations that did not require systemic steroids, resulting in a lower perceived rate. However, it is important to note that the severity of COPD in our study was no different between the two cohorts and that other studies resembling ours have reported similar rates of COPD exacerbation.

Anecdotal data suggest that many COPD patients who might otherwise qualify to receive *β*-blockers are not receiving them [9]. Data supporting the safety of perioperative *β*-blockers among COPD patients have significant clinical ramifications and imply a larger cohort of patients who might benefit from the cardio protective effects of these drugs. Results of this study add to the growing literature suggesting that *β*-blockers can be safely administered to COPD patients in specific clinical situations. van der Woude et al. showed that the use of Celiprolol, a *β*_1_ selective blocker, did not cause any changes in lung function or bronchoreactivity [26]. In another study COPD patients undergoing major vascular surgery and receiving *β*-blockers had a lower 30-day and long-term mortality than those who did not [27]. A retrospective analysis by Dransfield et al. suggested that *β*-blocker use among patients hospitalized with acute COPD exacerbations was associated with decreased in-hospital mortality [28].

Postoperative atrial fibrillation developed in approximately 15% of the patients in this study. The rate of atrial fibrillation between the *β*-blocker and no *β*-blocker groups was similar despite the fact that the former group has a higher prevalence of heart disease (38%* versus* 9%). This rate is also similar to the incidence reported after lung resection in the nontreatment group of the retrospective study done by Okita et al. [19–22].

It is worthwhile noting some other limitations to this study. It is observational by design, and patients were limited to a single metropolitan cancer center in the Northeastern United States. The fact that more patients receiving *β*-blockers had previously documented heart disease suggests possible confounding by indication. It is expected, however, that this would have biased the results towards more frequent cardiac and pulmonary complications in the *β*-blocker group but it did not. The dose of *β*-blockers used in our cohort was generally small, which may have influenced the rate of respiratory complications. Similarly, most patients with COPD had relatively mild airways obstruction. Finally, it is possible that the sample size was insufficient to detect true differences in the rate of COPD exacerbations.

In summary, this study suggests that perioperative *β*-blocker use among COPD patients undergoing lung resection surgery is not associated with deleterious pulmonary complications or length of hospital stay, ICU admission, or 30-day mortality. Randomized interventional trials involving COPD patients are needed to confirm these findings.

## Figures and Tables

**Table 1:** Clinical characteristics of patients that received perioperative *β*-blockers in comparison to those that did not. Data are expressed as the number of patients in each group.

Clinical variable (%)	*β*-blockers (*N* = 205)	No *β*-blockers (*N* = 315)	*p* value
Age >60 years	175 (85.4%)	222 (70.5%)	0.0001
Male sex	112 (54.6%)	130 (41.3%)	0.003
FEV1% <50	16 (7.8%)	26 (8.3%)	n.s.
DLCO% <50	18 (8.9%)	32 (10.3%)	n.s.
Prior history			
Asthma	6 (2.9%)	15 (4.8%)	n.s.
Cardiac disease	143 (69.8%)	113 (35.9%)	<0.0001
Chronic renal insufficiency	5 (2.5%)	4 (1.3%)	n.s.
Diabetes mellitus	27 (13.2%)	25 (7.9%)	n.s.
Smoking within 6 months	71 (34.8%)	127 (40.3%)	n.s.
Extent of lung resection			
Sublobar	48 (23.4%)	82 (26.0%)	n.s.
Lobectomy	133 (64.9%)	200 (63.5%)	n.s.
Bilobectomy/pneumonectomy	24 (11.7%)	33 (10.5%)	n.s.
Complications			
COPD exacerbation	11 (5.4%)	20 (6.4%)	n.s.
Respiratory failure	7 (3.4%)	10 (3.2%)	n.s.
Cardiac complication	18 (8.8%)	17 (5.4%)	n.s.
ICU transfer	12 (5.9%)	15 (4.8%)	n.s.
Death (30 d)	4 (2.0%)	1 (0.3%)	n.s.

**Table 2:** Incidence of patients that developed cardiac complications. Data are expressed as the number of patients in each group.

Cardiovascular complications	*β*-blockers (*N* = 205)	No *β*-blockers (*N* = 315)	*p* value
Acute coronary syndrome (%)	1 (0.5%)	0 (0 %)	n.s.
Atrial fibrillation (%)	29 (14.1%)	49 (15.6%)	n.s.
CHF (%)	1 (0.5%)	0 (0%)	n.s.
Other	3 (1.5%)	3 (0.9%)	n.s.
Composite cardiac endpoints	34 (16.6%)	52 (16.5%)	n.s.

**Table 3:** Specific *β*-blocker used among 205 patients.

	*β*-blockers	Number of patients (% of patients)
Cardioselective	Atenolol	11 (5%)
	Metoprolol	187 (91%)

Nonselective	Propranolol	1 (1%)
	Carvedilol	2 (1%)
	Labetalol	4 (2%)

**Table 4:** Clinical predictors of COPD exacerbations.

Variable	Univariate odds ratio	Univariate *p* value	Multivariate odds ratio	Multivariate *p* value
Age >60 years	3.06 (1.06–12.95)	0.04	2.50 (0.84–10.72)	0.11
Male sex	1.42 (0.69–2.99)	0.34		
Perioperative *β*-blocker given	0.84 (0.38–1.77)	0.65		
FEV1% <50	4.62 (1.83–10.77)	0.00	2.64 (0.87–7.38)	0.08
DLCO% <50	4.39 (1.82–9.90)	0.00	2.83 (0.98–7.57)	0.05
Asthma	1.79 (0.28–6.63)	0.48		
Cardiac disease history	1.71 (0.82–3.68)	0.15	1.69 (0.79–3.76)	0.18
Chronic renal insufficiency	1.99 (0.11–11.36)	0.56		
Diabetes mellitus	0.96 (0.22–2.83)	0.94		
Smoking within six months	1.18 (0.55–2.45)	0.66		
Extent of lung resection				
Sublobectomy (referent)	1.00 (—)	—		
Lobectomy	0.97 (0.43–2.40)	0.95		
Bilobectomy/pneumonectomy	0.84 (0.18–3.03)	0.80		

## Abbreviations (in Alphabetical Order)

ACS: Acute coronary syndrome
CHF: Congestive heart failure
COPD: Chronic obstructive pulmonary disease
DLCO%: Diffusing capacity for carbon monoxide, expressed as percent predicted
FEV1: Forced expiratory volume in one second
FEV1/FVC: Forced expiratory volume in one second and forced vital capacity
FVC: Forced vital capacity.
